# Clinical efficacy and safety of the combination of mesenchymal stem cells and scaffolds in the treatment of knee osteoarthritis: Protocol for systematic review and meta-analysis

**DOI:** 10.1097/MD.0000000000031638

**Published:** 2022-10-28

**Authors:** Qinglin Wu, Zuqing Wu, Zhifu Lu

**Affiliations:** a Department of Massage, Haikou Hospital of Traditional Chinese Medicine, Hainan, China; b Department of Orthopedics and Traumatology, Haikou Hospital of Traditional Chinese Medicine, Hainan, China.

**Keywords:** knee osteoarthritis, mesenchymal stem cells, meta-analysis, scaffolds

## Abstract

Mesenchymal stem cells (MSCs) injection combined scaffolds for knee osteoarthritis (OA) is a relatively new treatment for knee OA and has not yet gained popularity. So, the effectiveness, safety is worthy to be explored. We performed a protocol for systematic review and meta-analysis to evaluate the efficacy and safety of the combination of MSCs and scaffolds in the treatment of knee OA.

**Methods:**

A literature search was performed in October 2022 without restriction to regions, publication types or languages. The primary sources were the electronic databases of PubMed, EMBASE, Cochrane Library, Web of Science and the ClinicalTrials.gov. Risk of bias was assessed using the Cochrane Collaboration’s risk of bias tool for randomized controlled trials. Statistical analyses were performed utilizing Review Manager 5 (The Nordic Cochrane Center, Copenhagen, Denmark).

**Results:**

Visual analog scale score, Western Ontario and McMaster Universities Osteoarthritis Index, Lysholm knee scale and adverse events will be assessed.

**Conclusion:**

The systematic review will provide evidence to assess the effectiveness and safety of MSCs combined scaffolds for the treatment of knee OA.

## 1. Introduction

Knee osteoarthritis (OA) is a common disease associated with progressive deterioration of the cartilage and narrowing of the joint space.^[1–3]^ Epidemiological statistics show that the overall prevalence of primary OA in people aged more than 40 years is 46.3%, 41.6% for male and 50.4% for female and the number of knee OA is continually growing due to the aging population.^[4,5]^ Specifically, symptoms of OA include continuous chondrocyte cartilage damage, articular chondrocyte loss, subchondral microfracture, subchondral bone exposure, joint edge and subchondral bone hyperplasia.^[6]^ Clinically OA patients suffer from slowly developing joint pain, joint stiffness, joint swelling, decreased joint range of movement and joint deformity.

Currently, the treatment for knee OA is very limited. There are some conventional therapies for knee OA, including physiotherapy, nonsteroidal anti-inflammatory drugs, pain-relieving drugs, hyaluronic acid, platelet-rich plasma or corticosteroid-based intra-articular injections, traditional Chinese medicine and knee arthroscopic surgery.^[7–9]^ All the above-mentioned treatments can only relieve symptoms, but cannot repair cartilage. As OA worsens, total knee arthroplasty is needed.

Stem cells therapy is a milestone in regenerative medicine for OA treatment. Mesenchymal stem cells (MSCs) have self-renewal and multidirectional differentiation potential, and can exert therapeutic effects on various diseases through directed differentiation, regulation of immunity, anti-inflammatory, proangiogenesis, improvement of microenvironment and promotion of regeneration.^[10]^ MSCs have been used in the treatment of various diseases, such as premature ovarian failure, Parkinson’s disease, nervous system damage and amyotrophic lateral sclerosis.^[11,12]^ MSCs therapy could be applied for OA treatment and have shown encouraging results. The usage of MSC in combination with scaffolds is promising as a tool in the treatment of damaged tissues that have specific functions. We performed a protocol for systematic review and meta-analysis to evaluate the efficacy and safety of the combination of MSCs and scaffolds for the treatment of knee OA.

## 2. Methods

The proposed systematic review and meta-analysis will conform to the Preferred Reporting Items for Systematic Review and Meta-analysis Protocols.^[13]^ This protocol is registered with the International Prospective Register of Systematic Reviews (PROSPERO), registration number (CRD42021276811). Ethics application was not required as this study is based on published trials.

### 2.1. Literature-search strategy

A literature search was performed in October 2022 without restriction to regions, publication types or languages. The primary sources were the electronic databases of PubMed, EMBASE, Cochrane Library, Web of Science and the ClinicalTrials.gov. Two authors will independently draft and carry out the search strategy. The gray literature will be searched in databases such as OpenGrey. Articles will also be searched from the references of the retrieved studies. The key terms used for the search are “knee osteoarthritis,” “mesenchymal stem cells” and “scaffolds.” The search strategy used in PubMed is presented in Table 1.

**Table 1:** Search strategy for PubMed.

Serial number	Line
#1	“knee osteoarthritis”[Title/Abstract] OR “degenerative osteoarthropathy”[Title/Abstract] OR “knee deformity”[Title/Abstract]
#2	“mesenchymal stem cell”[Title/Abstract] OR “mesenchymal stromal cells”[Title/Abstract] OR “mesenchymal stromal cell”[Title/Abstract] OR “bone marrow mesenchymal stem cell*”[Title/Abstract] OR “bone marrow stromal cell”[Title/Abstract] OR “bone marrow stromal stem cells”[Title/Abstract] OR “multipotent bone marrow stromal cell”[Title/Abstract] OR “adipose derived mesenchymal stem cell”[Title/Abstract] OR “adipose tissue derived mesenchymal stem cell”[Title/Abstract] OR “adipose tissue derived mesenchymal stromal cells”[Title/Abstract] OR “adipose derived mesenchymal stromal cells”[Title/Abstract] OR “adipose derived mesenchymal stem cell”[Title/Abstract] OR “multipotent mesenchymal stromal cell”[Title/Abstract] OR “mesenchymal progenitor cell”[Title/Abstract] OR “wharton jelly cells”[Title/Abstract] OR “whartons jelly cells”[Title/Abstract]
#3	“scaffolds”[Title/Abstract] OR “tissue engineering”[Title/Abstract] OR “biological patch”[Title/Abstract]
#4	#1 and #2 and #3

### 2.2. Inclusion and exclusion criteria

**Figure 1.**
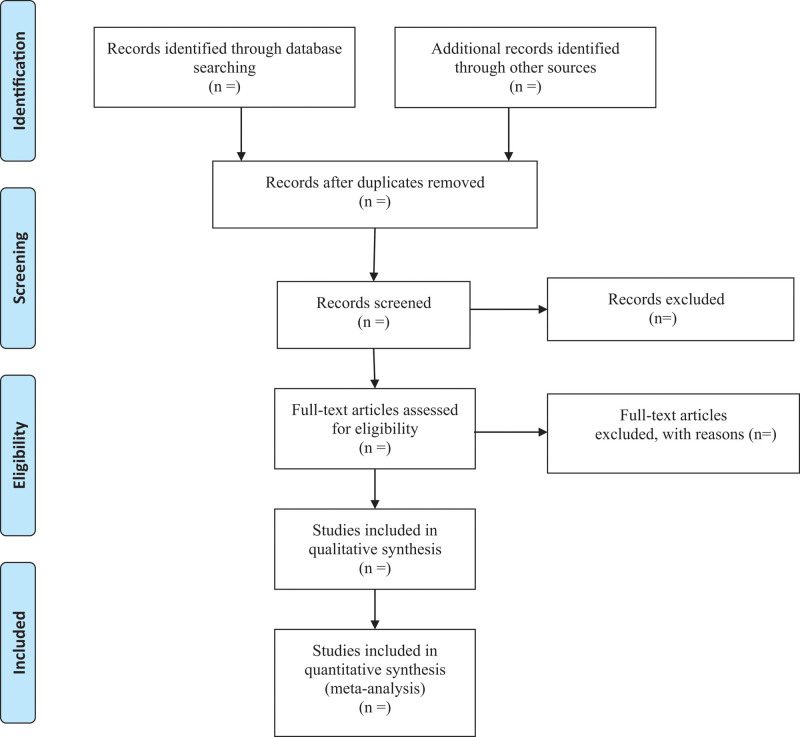
Flow diagram of study selection.

Studies were eligible for inclusion if: Randomized controlled trial on patients with knee OA; Diagnosis of knee OA was based on the clinical and radiological criteria defined by the American College of Rheumatology and illustrated degree of OA (Kellgren-Lawrence grade); Definition of MSCs combined scaffolds in the intervention group must meet the minimum standards set out in the International Society for Cytotherapy Consensus Statement and be administered by intra-articular injection; Editorials, letters to the editor, review articles, case reports and animal experimental studies were excluded. The selection process of eligible papers is shown in a PRISMA flow diagram (Fig. 1).

### 2.3. Data extraction

The above 2 authors independently recorded data from each study using a predefined data extraction form. Data on age, number of cases, follow-up, grade of OA, site of source, source (autologous or allogeneic), methods and timing of delivery, culture expansion, entity of cells, number of cells, control intervention, and concomitant treatment were collected. Entity of cell population was clearly evaluated based on a consensus statement. Outcome scales regarding pain and function were recorded for the following: visual analog scale, Western Ontario and McMaster Universities Osteoarthritis index,^[14]^ and Lysholm knee scale. In addition, Magnetic Resonance Observation of Cartilage Repair Tissue and Whole-Organ Magnetic Resonance Imaging Score on MRI were extracted to evaluate cartilage repair.

### 2.4. Assessment of risk of bias

Risk of bias was assessed using the Cochrane Collaboration’s risk of bias tool by 2 authors independently.^[15]^ The following factors were assessed: random sequence generation (selection bias), allocation concealment (selection bias), blinding of participants and personnel (performance bias), blinding of outcome assessment (detection bias), incomplete outcome data (attrition bias), selective reporting (reporting bias), and other bias. According to these items, each of included studies was scored as to be at low, unclear, or high risk of bias. Disagreements were resolved by discussion and assessed by kappa value.

### 2.5. Statistical analysis

For the meta-analyses, pooled estimates of effect sizes were calculated using a random effects model for the primary outcomes of self-reported pain and physical function, and cartilage structural changes. Standardized mean differences and 95% confidence interval were used to assess outcome improvement from baseline to the longest follow-up time point, comparing subjects receiving MSCs combined scaffolds and controls. For outcomes measured with different assessment tools, such as self-reported physical function and cartilage quality, individual studies in the meta-analyses were grouped according to scoring metric. The magnitude of the standardized mean differences was assessed according to Cohen’s d estimate. Briefly, <0.5, 0.5 to 0.8, and > 0.8 correspond to small, medium, and large effect sizes, respectively. Study heterogeneity was assessed with I-squared tests.

Subgroup analysis will be performed to reduce the heterogeneity and ensure the accuracy of results. Statistical analyses were performed utilizing Review Manager 5 (The Nordic Cochrane Center, Copenhagen, Denmark).

## 3. Discussion

Current studies show that MSCs have the following functions: interacting with the immune system and promote the immuno-regulation; migrating to the injury to enhance the tolerance of peripheral tissues, inhibit the release of inflammatory factors, promote the repair of injured tissues and increase the activity of injured cells; having great potential of multidirectional differentiation and reproductive activity; and secreting a variety of cytokines, such as transforming growth factor-β1, hepatocyte growth factor, fibroblast growth factor and vascular endothelial growth factor, which have an effect on anti-inflammatory, anti-apoptosis, anti-fibrosis, pro-angiogenesis, pro-mitosis, pro-wound healing, etc.^[16,17]^ Therefore, the application of MSCs could be applied for OA treatment. Recently, extracellular matrix-based tissue-engineered is a promising approach to repairing bone defects, and the seed cells are mostly MSCs.^[18,19]^ However, it remains unclear which exact pathways and factors that participate in the mechanism of MSCs to repair the damaged knee joint cartilage. Further basic and clinical researches are still required.

## Author contribution

**Conceptualization:** Zuqing Wub.

**Data curation:** Zuqing Wub.

**Writing – original draft:** Qinglin Wua.

**Writing – review & editing:** Zhifu Lu.
